# Mitral regurgitation secondary to ventricular remodelling post myocardial infarction – an echocardiographic study

**DOI:** 10.1186/1753-6561-6-S4-P24

**Published:** 2012-07-09

**Authors:** N Thakur, A Ionac

**Affiliations:** 1 “Victor Babes” University of Medicine and Pharmacy, Timisoara, Romania; 2 Timisoara Institute of Cardiovascular Medicine, Romania

## Introduction

To evaluate the incidence and mechanism of mitral regurgitation [MR] as a complication in the evolution of myocardial infarction [MI]. Patient population: 95 patients diagnosed on the basis of clinical, electrocardiographic and coronary-angiographic data with coronary artery disease [CAD] with a history of MI with haemodynamically significant MR (>/=grade II).

• All patients were treated in accordance with standard therapy for CAD and heart failure

• 56 Patients with PTCA of the obstructed coronary artery

• 28 Patients treated with CABG out of these 19 patients with mitral valvuloplasty

Criteria for exclusion from the study: patients with organic MR (mitral valve modified as a result of rheumatic fever, infectious endocarditis or degeneration) or with myxomatous mitral valve prolapse.

## Methods

Transthoracic echocardiography [TTE] for all patients and transoesophageal echocardiography for 75 patients.

## Results

Sixty eight patients had grade II MR and twenty seven grade III MR. The aetiology of MR was:

• Papillary muscle rupture – 1 patient

• Papillary muscle ischaemia (inferior or posterior MI) – 15 patients

• Dilatation and remodeling of left ventricle [LV] – 58 patients (13 out of these 58 patients had LV aneurysms)

• Mixed mechanism (papillary muscle ischaemia and LV dilatation) – 21 patients

## Conclusions

MR appears frequently in the evolution of patients with MI, even in patients subjected to a standard line of treatment. Echocardiography is a very important tool for the evaluation of severity and more importantly, that of aetiology. Understanding of the mechanism of MR offers the possibility to find a correct treatment option and where necessary, an optimal surgical intervention.

